# First-trimester fetal growth restriction and the occurrence of miscarriage in rural Bangladesh: A prospective cohort study

**DOI:** 10.1371/journal.pone.0181967

**Published:** 2017-07-21

**Authors:** Harunor Rashid, Enbo Ma, Farzana Ferdous, Eva-Charlotte Ekström, Yukiko Wagatsuma

**Affiliations:** 1 International Centre for Diarrheal Disease Research, Bangladesh (icddr,b), Dhaka, Bangladesh; 2 Graduate School of Comprehensive Human Sciences, University of Tsukuba, Tsukuba, Japan; 3 Department of Clinical Trial and Clinical Epidemiology, Faculty of Medicine, University of Tsukuba, Tsukuba, Japan; 4 Department of Women’s and Children’s Health, International Maternal and Child Health, Uppsala University, Uppsala, Sweden; Colorado State University, UNITED STATES

## Abstract

Fetal growth restriction in early pregnancy increases the risk of adverse pregnancy outcome, which has a significant social and psychological impact on women. There is limited information related to community-based study to evaluate early indicators related to miscarriage. The aim of this study is to examine the relationship between fetal growth restriction, measured by ultrasound crown-rump length (CRL), and subsequent occurrence of miscarriage in pregnant women in rural Bangladesh. The study was conducted within the Maternal and Infant Nutrition Interventions Trial in Matlab (MINIMat study), Bangladesh. A total of 4436 pregnant women were enrolled in the study when they were at less than 14 gestational weeks. The expected CRL was determined based on an established growth curve of gestational age and CRL, and deviation from this curve of CRL was expressed as a z-score. After identifying related covariates, the multiple Poisson regression model was used to determine the independent contribution from the CRL to miscarriage. A total of 3058 singleton pregnant women were included in analyses, with 92 miscarriages and 2966 continued pregnancies. The occurrence of miscarriages was significantly higher in the smaller categories of CRL z-score after adjustments for maternal age, parity, early pregnancy BMI, gestational age at CRL measurement and socioeconomic status (adjusted relative risk [95% confidence interval]: 1.03 [1.02–1.05] for less than -2 z-score). In a rural Bangladesh population, smaller than expected CRL for the gestational age was related to subsequent miscarriage. Ultrasound biometry information together with careful clinical assessment should provide much needed attention and care for pregnant women.

## Introduction

Spontaneous miscarriage, one of the most common pregnancy complications, is not only associated with morbidity or mortality [1], but also has a significant social and psychological impact on women [2]. The incidence of spontaneous miscarriages in pregnancies was reported to be as high as 15%, and at least 80% of those occurred in the first trimester of pregnancy [3].

Chromosomal abnormalities followed by uterine malformations are the most common etiologies of spontaneous miscarriage in early pregnancy [4]. Advanced maternal age, smoking, alcohol consumption, drug abuse, vaginal bleeding, and previous history of miscarriages are the commonly reported risk factors of spontaneous miscarriages [5–8]. Early growth restriction, fetal heart rate, gestational sac diameter, and yolk sac diameter have been used as early predictors of subsequent miscarriage [9, 10]. Conversely, the crown-rump length (CRL) measurement is also clinically used for predicting adverse pregnancy outcomes of threatened abortion or risk of spontaneous miscarriage in early pregnancy [11–13]. Most of these studies were hospital-based and conducted in developed countries with a small number of selected populations. Two population-based studies, conducted in the United Kingdom and Sweden, examined the association between maternal risk factors and miscarriages [14, 15]. However, these studies did not examine any ultrasound parameters for predicting subsequent miscarriage in early pregnancy.

Hospital-based studies may also be limited in their ability to consider the reproductive outcomes among a general healthy population. It requires a routine data collection system that can cover both the full range of miscarriages and link to individual-based data [14]. Such data, however, are scarce from developing countries. Moreover, only a limited number of community-based studies have been conducted to evaluate early ultrasound indicators related to miscarriage in developing countries, where malnutrition is prevalent and socioeconomic status differs greatly. Therefore, in this population-based prospective study, carried out in rural Bangladesh, we aimed to examine whether smaller than expected CRL is a risk for subsequent miscarriage.

## Materials and methods

This study was conducted as part of a large-scale randomized trial of nutrition interventions in pregnancy: the Maternal and Infant Nutrition Interventions Trial in Matlab (MINIMat) study (trial registration: isrctn.org identifier: ISRCTN16581394). Women in the MINIMat study were recruited from November, 2001 to October, 2003 in the Matlab Health and Demographic Surveillance System area of the International Centre for Diarrheal Disease Research, Bangladesh (icddr,b). Details of the study location and the trial are described elsewhere [16].

Pregnancy urine tests were offered to every woman who reported to the Community Health Research Workers (CHRWs) that her last menstrual period (LMP) was at least 2 weeks overdue, or that she was pregnant. The LMP date was determined by recall during the pregnancy identification interview during routine monthly household visits. Women with positive pregnancy tests were invited to join the MINIMat study and their LMP dates were recorded. They were then invited to visit a nearby icddr,b clinic for evaluation of viable fetus and measurement of gestational age (GA) by ultrasound examination. Some women came for ultrasound examination before the 5th week of their GA, thus crown rump length (CRL) could not be measured. These women were either rescheduled or given another date for their ultrasound examination. To participate in the MINIMat study, the inclusion criteria for a pregnant woman were: 1) viable fetus, 2) GA less than 14 weeks, and 3) women who gave their consent.

**Fig 1:**
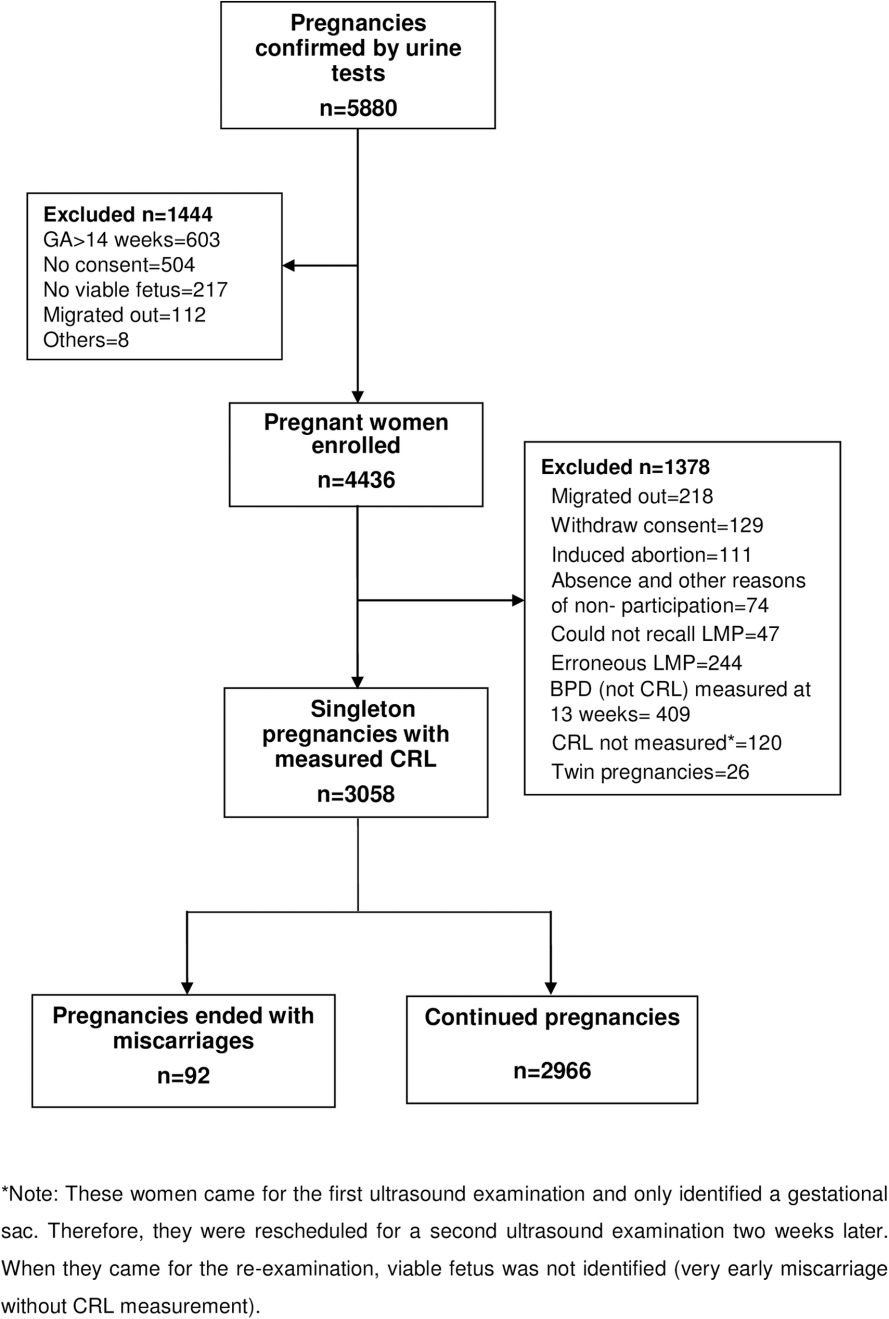
Flow chart of study subjects.

A total of 5880 women were identified as eligible for the MINIMat study. Of these women, 1444 were excluded because of migration out of the study area, refusal to participate, having a fetus whose GA exceeded the limits for the study, no longer having a viable fetus (determined by ultrasound), and other reasons (Fig 1). A total of 4436 women were enrolled to follow up with ultrasound examination during clinic visits at 14, 19, and 30 weeks of pregnancy. Of the 4436 women, 218 were excluded from the study for reasons of migration, 129 for withdrawal of consent, 111 for induced abortion, 74 for absence, and other reasons. For this current analytical study of miscarriage, additional inclusion criteria pregnant women with successful CRL measurement and singleton fetus. A further 291 women had to be excluded due to missing (could not recall) LMP dates (n = 47) or erroneous LMP information (n = 244). We defined the recalled LMP as erroneous if the ultrasound-estimated LMP and recalled LMP had a difference more than 21 days. Additionally, 409 women had to be dropped from the analyses because their CRLs were not measured but biparietal diameter [BPD] was measured at 13 gestational weeks. Furthermore, 26 twin pregnancies were excluded. Of 4436 MINIMat eligible women, a total of 236 (5.3%) miscarriages occurred, but 120 early miscarriages were identified at the time of ultrasound examination during the rescheduled clinic visits. For these women only the gestational sac could be identified at initial ultrasound examination, thus, a second ultrasound examination was scheduled 2 weeks later (minimum duration). At reexamination, a viable fetus could not be identified (very early miscarriage without CRL measurement) and CRL could not be measured. Thus, they were excluded from the current analysis. Although, CRL was measured in 116 miscarried fetuses, of these 24 were excluded (6 could not recall LMP date, 15 had erroneous LMP information, and 3 were twin pregnancies). Finally, 3058 singleton pregnant women were included in the analysis (Fig 1). Of those, 2966 continued with viable pregnancies until delivery and 92 had miscarriages.

Four ultrasound machines (SSA 320A, Justavision-200, Toshiba, Tokyo, Japan) with 3.5 MHz standard convex probes were used for the fetal biometry measurements including CRL. Three measurements were taken and the average value was used for analysis. The intra-observer variation and quality control of ultrasound measurements were described elsewhere [17].

Pregnant women were interviewed monthly to identify pregnancy outcomes such as spontaneous abortion, induced abortion, stillbirth, live birth, and survival in infancy (The Final Disposition and Birth Forms were provided as “S1 Supporting Document” and “S2 Supporting Document”). Trained female field workers prospectively collected the outcome information. A study physician reviewed each outcome, and miscarriages were confirmed at the icddr,b clinics. Miscarriage was defined as an unintended loss of fetus before the first 20 weeks of gestation as determined by the reported LMP. Stillbirth was defined as birth of a dead fetus at the gestation week of 20 or more [18]. Live birth was defined as birth of a fetus with any sign of viability. Parity is the number of live and/or deceased births before the current pregnancy [19]. Socioeconomic status was assessed by generating scores through principal-components analysis based on household assets, housing structure, land occupation, and income. These scores were then indexed into quintiles, where 1 represents the poorest and 5 the richest [20]. Height and weight measurements of the pregnant women were taken at the time of enrollment, between 6 and 13 week of gestation. Body Mass Index (BMI) (kg/m^2^) was categorized as either under nutrition (<18.5), normal (18.5–24.9), or overweight (≥25).

The observed CRL was measured during ultrasound examination at enrollment (between 6 and 13 weeks of gestation). The expected CRL was determined based on an international reference of GA and CRL [21]. The difference between the observed and expected CRL was calculated based on the formula: observed value minus expected value divided by standard deviation (SD), and expressed as a z-score. The CRL z-scores were compared between the miscarriage and viable pregnancy groups. A grade of 0 on the CRL z-score means that the CRL is the same as expected for the corresponding GA. A positive or negative CRL z-score indicates that CRL is above or below expected for the corresponding GA. The CRL z-score was categorized in different recommended values as follows: 1^st^ (-1 or more), 2^nd^ (-2 to less than -1), 3^rd^ (-3 to less than -2), and 4^th^ (less than -3).

Maternal factors and CRL z-score were compared between miscarriage and viable pregnancy groups. For continuous variables, independent t-test was performed to compare the mean between the 2 groups. Chi-square test was used for comparing proportions between categorical variables. Multiple Poisson regression model was used to determine the independent contribution from the CRL to miscarriage by controlling for socioeconomic status, maternal age, parity, gestational age at CRL measurement, and BMI. The strength of association was expressed by adjusted relative risk (aRR) and 95% confidence interval (CI). Associations were considered significant at a *P* value of < 0.05. Statistical analysis was performed using SPSS version 22 (IBM, Armonk, NY, USA).

The study was conducted according to the guidelines laid down in the Declaration of Helsinki. All procedures involving human subjects were approved by the ethical review committees of icddr,b and Uppsala University. Written informed consent was obtained from each participant.

## Results

The mean gestational age at CRL measurement was 9.4 ± 1.5 weeks. The mean maternal age at enrollment was 25.8 ± 5.8 years (range 14–50 years). One third of the women (33.7%) were nulliparous. Only 67.9% of the women had attended school. The mean of early pregnancy BMI was 20.2 ± 2.6 kg/m^2^ and 27.4% of women were underweight (Table 1).

**Table 1:** Characteristics of study participants (n = 3058).

Variables		n (%)
Gestational age (wk) at CRL measurement [mean ± SD]^b^		[9.4 ± 1.5]^a^
Maternal age [Mean ± SD]		[25.8 ± 5.8]^a^
Age group, y		
	14–19	470 (15.4)
	20–24	895 (29.3)
	25–29	872 (28.5)
	30–34	560 (18.3)
	≥35	261 (8.5)
Parity		
	0	1030 (33.7)
	≥1	2028 (66.3)
Educational status		
	Illiterate	983 (32.1)
	Literate	2075 (67.9)
BMI, kg/m^2^ [mean ± SD]		[20.2 ± 2.6]^a^
BMI, kg/m^2^		
	<18.5	838 (27.4)
	18.5–24.9	2036 (66.6)
	≥25.0	184 (6.0)
Socioeconomic quintile		
	1^st^ (poorest)	593 (19.4)
	2^nd^	608 (19.9)
	3^rd^	605 (19.8)
	4^th^	618 (20.2)
	5^th^	634 (20.7)

Abbreviation: BMI; body mass index

^a^ Values are given as [mean ± SD]

^b^Cohort inception; occurred at the time of CRL successful measurement.

The mean cohort follow-up time for the miscarried pregnant women was 3.91±2.6 (range, 0–12) weeks and for continued pregnant women was 29.71±2.6 (range, 15–37) weeks. The distribution of gestational age at CRL measurement and follow-up time of the study participants in the cohort shows in “S1 Table”. The mean z-score of CRL was significantly lower in the miscarriage group of women compared with continued viable pregnancy group (-1.43 vs. -0.80, *P* = 0.030). The rate of miscarriages was almost double among the women carrying fetuses with smaller than expected CRL for GA (negative z-scores) compared with women carrying fetuses with larger CRL (3.6%[72/1983] vs. 2.1%[20/1075]; *P* = 0.003). The occurrence of miscarriages was significantly more in the smaller categories of CRL z-score (2^nd^, 3^rd^, and 4^th^) compared with the 1^st^ category (z-score -1 or more; *P* = 0.004). The mean maternal age was significantly older in the miscarriage group than in the continued viable pregnancy group (27.4 vs. 25.7, *P* = 0.008). The occurrence of miscarriages was more likely to be higher among older women aged ≥ 35 years (*P* = 0.032). The number of overweight women was higher in the miscarriage group than in the continued viable pregnancy group (12.0 vs. 5.8, *P* = 0.021). Parity and food supplementation were not significantly different between the 2 groups (Table 2).

**Table 2:** Comparisons between miscarriage and continued pregnancy groups (n = 3058).

Variables		Pregnancies with miscarriage n = 92 (%)	Continued Pregnancies n = 2966 (%)	P value
CRL z score [mean ± SD]		[-1.43 ± 2.7]^a^	[-0.80 ±2.7]^a^	0.030^b^
CRL z-score categories				
	1st (-1 or more)	31 (2.0)	1534 (98.0)	0.004^c^
	2nd (-2 to less than -1)	19 (3.8)	484 (96.2)	
	3rd (-3 to less than -2)	20 (5.1)	375 (94.9)	
	4th (less than -3)	22 (3.7)	573 (96.3)	
Gestational age (wk) at CRL measurement [mean ± SD]^d^		[8.88 ± 1.4]^a^	[9.46 ±1.5]^a^	<0.001^b^
Maternal age [mean ± SD]		[27.4 ± 6.6]^a^	[25.7±5.8]^a^	0.008^b^
Maternal age, y				
	14–19	12 (2.6)	458 (97.4)	0.032^c^
	20–24	21 (2.3)	874 (97.7)	
	25–29	25 (2.8)	847 (97.2)	
	30–34	18 (3.2)	542 (96.8)	
	≥35	16 (6.1)	245 (93.9)	
Parity				
	0	31 (3.0)	999 (97.0)	0.539^c^
	≥1	61 (3.0)	1967 (97.0)	
BMI, kg/m2				
	<18.5	18 (2.1)	820 (97.9)	0.021^c^
	18.5–24.9	63 (6.0)	1973 (94.0)	
	≥25.0	11 (3.1)	173 (96.9)	
Socioeconomic quintile				
	1st (poorest)	26 (4.4)	567 (95.6)	0.263^c^
	2nd	16 (2.6)	592 (97.4)	
	3rd	15 (2.5)	590 (97.5)	
	4th	19 (3.1)	599 (96.9)	
	5th	16 (2.5)	618 (97.5)	

Abbreviation: BMI; body mass index, CRL; crown rump length

^a^ Value are given as [Mean ± SD]

^b^ *P* value by t-test

^c^ *P* value by chi-square test

^d^ Cohort inception; occurred at the time of CRL successful measurement.

After adjusting for maternal age, parity, early pregnancy BMI, GA at CRL measurement, and socioeconomic quintile, the smaller CRL categories continued to have a greater number of miscarriages. The adjusted relative risks (aRR) of miscarriage (expressed as 95%CI) were 1.02 (1.00–1.04), 1.04 (1.01–1.06), and 1.03 (1.01–1.05) in the 2^nd^, 3^rd^, and 4^th^ categories of CRL z-score, respectively in comparison with the 1^st^ category of CRL z-score (Table 3). When the cut-off of -2 z-score was used, aRR (95%CI) was 1.03 (1.02–1.05). The risk of miscarriages was significantly higher in women aged ≥ 35 years than those aged 25–29 years (*P* = 0.021). The occurrence of miscarriages was higher in the poorest quintile of women than the richest (*P* = 0.030; Table 3).

**Table 3:** Multivariate analysis in prediction of miscarriages (n = 3058).

Variables		cRR (95% CI)	*P* value	aRR^a^ (95% CI)	*P* value
CRL z-score category					
	1^st^ (-1 or more)	ref		ref	
	2^nd^ (-2 to less than -1)	1.90 (1.09–3.31)	0.023	1.02 (1.00–1.04)	0.009
	3^rd^ (-3 to less than -2)	2.64 (1.49–4.68)	0.001	1.04 (1.01–1.06)	<0.001
	4^th^ (less than -3)	1.94 (1.09–3.47)	0.025	1.03 (1.01–1.05)	<0.001
Maternal age, y					
	14–19	0.89 (0.44–1.78)	0.738	0.98 (0.96–1.01)	0.316
	20–24	0.81 (0.45–1.47)	0.493	0.99 (0.97–1.00)	0.385
	25–29	ref		ref	
	30–34	1.13 (0.61–2.08)	0.707	1.00 (0.98–1.02)	0.501
	≥ 35	2.21 (1.16–4.21)	0.016	1.03 (1.00–1.06)	0.021
Parity					
	0	1.00 (0.64–1.55)	0.998	1.01 (0.99–1.03)	0.074
	≥1	ref		ref	
BMI (kg/m2)					
	<18.5	0.69 (0.41–1.17)	0.166	0.99 (0.97–1.00)	0.161
	18.5–24.9	ref		ref	
	≥25.0	1.99 (1.03–3.85)	0.041	1.02 (0.99–1.06)	0.130
Socioeconomic quintile					
	1^st^ (poorest)	1.77 (0.94–3.34)	0.077	1.02 (1.00–1.04)	0.030
	2^nd^	1.04 (0.52–2.11)	0.904	1.00 (0.98–1.02)	0.573
	3^rd^	0.98 (0.48–2.00)	0.960	1.00 (0.98–1.01)	0.856
	4^th^	1.23 (0.62–2.41)	0.555	1.00 (0.98–1.02)	0.429
	5^th^	ref		ref	

Abbreviation: aRR; adjusted relative risk; cRR; crude relative risk; CI; confidence interval; ref; reference category

^a^ Adjusted by maternal age, parity, gestational age at CRL measurement, BMI and socioeconomic quintile

## Discussion

The present population-based study shows that smaller CRL is related to miscarriages in early pregnancy before 20 weeks. In our study, nearly half of the pregnancies that subsequently miscarried showed a CRL measurement significantly lower than expected for the GA. Our study shows that the risk of miscarriage increases in small CRL categories. Women with a fetus of smaller than expected CRL had nearly twice the risk of miscarriage. These 2 groups of women, however, had significant differences between socio-economic strata, age, and nutritional status. In our study, advanced maternal age and poor socioeconomic status were also identified as potential risk factors for miscarriage.

Previous hospital-based studies have shown an association between a smaller than expected CRL and the increased probability of subsequent miscarriage [9, 10, 13]. A hospital-based study in London showed that the pregnancies with a CRL smaller than expected were more likely to be at risk for miscarriages [11]. Another hospital-based study in Egypt reported that approximately 60% of pregnancies that ended in subsequent miscarriage had smaller than expected CRL [12]. The present study shows that the risk of miscarriage increases with the advancing negative CRL z-score categories.

In women who have conceived naturally, it is assumed that ovulation occurs 14 days after their LMP. The difference between observed and expected fetal size may be due to the timing of ovulation and smaller than expected CRL and, therefore, reflects a delayed conception in relation to the LMP rather than a true fetal growth delay [22]. By considering these issues, the present study aimed to identify accurate LMP dates through a strong surveillance system and confirmation by study physicians at clinics.

Maternal age was an important variable in the prediction of miscarriage. The risk of miscarriage was increased with increasing maternal age. It is well documented that the majority of early fetal deaths are due to chromosomal abnormalities and that there is an exponential increase of the risk for fetal trisomy with increasing maternal age [6]. However, in the present study we did not have appropriate data to prove a correlation between chromosomal abnormalities and spontaneous miscarriage. Further community-based studies are required in order to understand the clinical and biological phenomena of spontaneous miscarriage. A study done in the United Kingdom showed that, in comparison to women aged 25–29 years, the occurrence of miscarriage sharply increased among women aged 35 years or more [4]. The results of the present study support these findings, suggesting that, for women in rural Bangladesh, increased maternal age is a significant risk factor for miscarriage. Miscarriages were significantly higher in the poor socioeconomic group of women. A similar finding was also observed in the United States [7].

A previous study conducted in the United Kingdom showed that low BMI was a risk factor for miscarriage [14], however a Finish study showed that both low and high BMI were risk factors for miscarriage in early pregnancy [23]. In the present study, univariate analysis shows that BMI is related to miscarriage. After adjusting for maternal age and parity, however, BMI was not associated with miscarriage. This might be due to the small percentage of women in the overweight group of our study, and thus the limited statistical power.

This study has several strengths. Firstly, it showed the incidence of natural phenomenon of miscarriage in a healthy population in which trained research members, using a strong surveillance system, followed up on the outcome of each pregnancy. Secondly, for quality assurance, an independent team of data collectors randomly selected 5% of the sample and repeated the interviews and measurements and compared the data with the interviews.

There are some limitations to this study. This study does not report some of the important factors that may influence early miscarriage such as history of miscarriage, vaginal bleeding, abdominal pain, non-steroidal anti-inflammatory drugs use, maternal diabetes, and hypertension. Of the women with CRL measurement, spontaneous miscarriage rate was 3% (92 of 3058) in this study, which is not generalizable as a universal miscarriage rate. In this study, the rate of spontaneous miscarriage was less because the study had a narrow window of CRL measurement (GA of 6–13 weeks). CRL cannot be measured before 6 weeks of gestational age, so the present study could not capture pregnancy losses before 6 weeks. Nevertheless, this study provides an opportunity for clinicians to counsel women with smaller CRL at their early stage of pregnancy. Another limitation was that this study could not measure the CRL of all the participant’s fetuses. This study focused on miscarriages with measured CRL and did not cover all miscarriages. Therefore, early miscarriages (occurring before 6 weeks) could not be discussed in the present study.

## Conclusions

This study suggests that, in rural Bangladesh, smaller CRL is associated with the occurrence of subsequent miscarriage. The CRL is usually measured at the first consultation in health facilities, especially in developing countries. Ultrasound biometry information together with careful clinical assessment should help provide much needed attention for pregnant women. Further studies are required to identify related risks of first-trimester growth restriction and miscarriage in developing countries.

## Supporting information

S1 Dataset Analysis file for miscarriage 3058. (XLSX)

S1 Supporting Document Final disposition form. (PDF)

S2 Supporting Document Birth form. (PDF)

S1 Table Distribution of gestational age at CRL measurement and follow-up time of the study participants in the cohort, n = 3058. (PDF)
