# Supplementary material for: First-trimester fetal growth restriction and the occurrence of miscarriage in rural Bangladesh: A prospective cohort study
Source: PLoS One. 2017 Jul 21;12(7):e0181967. doi: 10.1371/journal.pone.0181967 (PMC5521847; doi:10.1371/journal.pone.0181967)
Supplement: S1 Supporting Document — (PDF) [file pone.0181967.s002.pdf]

ICDDR,B: Centre for Health and Population Research.

Combined Interventions to Promote Maternal and Infant Health – a study in MINIMAT

Final Disposition Form

মহিলার নাম :_________________________ RID: |___|___|___|___|___|___|___|___|___|___|
(Name of Woman)

ব্লক (Block): A B C D____|____| CID: |___|___|___|___|___|___|___|___|___|___|

Village Name / Code:____|____|____|____|

ইন্টারভিউয়ারের নাম / কোড (Interviewer's Name/Code): __________________________________|____|____|____|

ইন্টারভিউয়ের তারিখ (Interviewer's date): ________/________/200______

F01 Final Disposition:

F01a. Follow-up completed ……………………………………………………………………………………1

Date of pregnancy outcome ………………………………………………________/___ _____/______

Type of pregnancy outcome ………………………… Abortion……………..1 [Stop]

Miscarriage…………..2 [Stop]

Still birth …………….3 [Stop]

Child status at follow-up completion…….. Alive and 24 months old……1[Stop]

Dead………………………...2

Date of child death ………………………………………………….. ________/________/______

F01b. Women died before pregnancy outcome …………………………………………….. 2

Date of death of women …………………………………………… ________/________/______

Describe events leading to death in box below ………………….… |_______|________|_______|

F01c. Women/Child out-migrated/Left study area for delivery…………………………….. 3

Date she left study area ……………………………………………… _______/_______/______

Destination: ____|________|_______|

F01d. Women/Child refused to continue in the study ……………………………………… 4

Date she refused ……………………………………………… _______/_______/______

Reasons given: ____|________|_______|

____|________|_______|

F01e. Women/Child is too sick to continue in the study ……………………………….. 5

Date she became sick ……………………………………………… _______/_______/______

Describe her illness in the box below____________________________________|________|_______|

F02a. Last study contact with the women at C30, H30, H34, H38, 1m, 2m, 3m, 4m, 5m, 6m, 7m, 8m, 9m, 10m, 11m, 12m, 15m, 18m, 21m, 24m.

F02b. Date of last contact………………………………………… _________/_________/_________
