# Supplementary material for: First-trimester fetal growth restriction and the occurrence of miscarriage in rural Bangladesh: A prospective cohort study
Source: PLoS One. 2017 Jul 21;12(7):e0181967. doi: 10.1371/journal.pone.0181967 (PMC5521847; doi:10.1371/journal.pone.0181967)
Supplement: S2 Supporting Document — (PDF) [file pone.0181967.s003.pdf]

International Centre for Diarrhoeal Disease Research, Bangladesh

Combined Interventions to Promote Maternal and Infant Health – MINIMAT

Birth Form

মহিলার নাম (Name of Woman):____________________________ CID: |___|___|___|___|___|___|___|___|___|

বক (Block): A B C D|____| RID: |___|___|___|___|___|___|___|___|___|___|

গ্রামের নাম/কোড (Village Name / Code: ____________________|____|____|____|

বাড়ির নাম / কোড (Bari Name/Code): ______________________|____|____|____|

বাচ্চার নাম (Name of the child): ________________________ RID: |___|___|___|___|___|___|___|___|___|___|

প্যারামেডিকের নাম/কোড (Paramedic Name/Code)______________________|____|____|

বাচ্চা হওয়ার খবর কবে পাওয়া গেছে? (Date of birth notification): ________/________/________

বাচ্চা হওয়ার খবর কিভাবে পাওয়া গেছে? (Source of birth notification):

- বাচ্চার পরিবার নোটিফিকেশন কার্ড পাঠিয়েছেন (Notification card sent by family) 1
- বাচ্চার পরিবার খবর পাঠিয়েছেন (Information sent by family) 2
- মাঠকর্মী খবর এনেছেন (Information obtained by field staff).. 3
- আই সি ডি ডি আর,বি সাব-সেন্টার বা হাসপাতালে বাচ্চা হয়েছে (Birth in ICDDR,B sub-centre/hospital) 4

জন্মের পর অসুস্থ বাচ্চা নিয়ে অন্য হাসপাতাল হতে সরাসরি সাব-সেন্টার / হাসপাতাল (After child birth in centre/hospital)..5

প্রাপ্ত খবর অনুযায়ী বাচ্চা কোথায় হয়েছে? (Reported location of birth)

- মতলবে বাড়ীতে (Home in Matlab)... 1
- আই সি ডি ডি আর,বি হাসপাতালে (ICDDR,B hospital).. 2
- আই সি ডি ডি আর,বি সাব-সেন্টারে (ICDDR,B sub-centre)... 3
- মতলবের ভিতরে-অন্য হাসপাতাল/ক্লিনিকে (Inside Matlab –other hospital/clinic)____________________4
- মতলবের বাইরে-হাসপাতাল/ক্লিনিকে (Outside Matlab – hospital/clinic) 5
- মতলবের বাইরে-বাড়ীতে (Outside Matlab – home)... 6
- মতলবের বাইরে-জানা নাই (Outside Matlab – unknown)... 7

প্রাপ্ত খবর অনুযায়ী বাচ্চা জন্মের তারিখ (Reported birth date): ________/________/________

ইন্টারভিউয়ের তারিখ<br>**Date of Interview**	সম্পূর্ণ হয়েছে<br>**Completed**	রাজী নন<br>**Refused**	বাসা ছেড়ে চলে গেছেন<br>**Out-migrated**	মহিলা বাসায় ছিলেন না<br>**Woman not at home**	অসম্পূর্ণ<br>**Incomplete**	মহিলা এখনও ফিরেননি<br>**Woman not returned yet**
1. ____/____/200	1	2	3	4	5	7
2. ____/____/200	1	2	3	4	5	7
3. ____/____/200	1	2	3	4	5	7

ইন্টারভিউ শুরু করার সময়: ___ ___: ___ ___ am/pm   ইন্টারভিউ শেষ করার সময়: ___ ___ : ___ ___am/pm

জমজ বাচ্চা (Twin child): না No (1) / হ্যাঁ Yes (2)   জমজ হলে বাচ্চার সংখ্যা (If twin, child number): 1 / 2

[জমজ বাচ্চা বা তিনটি বাচ্চার ক্ষেত্রে প্রতিটি বাচ্চার জন্যে আলাদা ফর্ম পুরণ করুন]

(In case of Twin/Triplet births, complete a separate form for each child)

Checked by: ____ **Date:** _______/_______/_______

Edited by: ____ **Date:** _______/_______/_______

Data entered by: ___________________________________ **Date:** _______/_______/_______

মা যদি খুব অসুস্থ হয় তাহলে তাকে যত তাড়াতাড়ি সম্ভব রেফার করুন। সেই ক্ষেত্রে এই ফর্মটি পরে পুরণ করতে হবে। মাকে রেফার করার জন্যে সাহায্য করুন।

[If the mother is very sick - please refer her immediately. The form can be completed later. Please help her with transportation]

মায়ের অংশ (Maternal):

MB01. আপনার কখন প্রসবের ব্যথা উঠেছিল? When did your labour pain start?

তারিখ (Date):.................. ______/______/______

সময় (Time): ....|____|____| am / pm

MB02. আপনার কখন পানি ভেঙেছিল? When did your water break?

তারিখ (Date):.................. ______/______/______

সময় (Time): ....|____|____| am / pm

MB03. আপনার কখন সন্তান প্রসব হয়েছিল? When was your baby born?

তারিখ (Date):.................. ______/______/______

সময় (Time): ....|____|____| am / pm

MB04. আপনার বাচ্চার জন্ম কোথায় হয়েছে? Where was the baby born?

বাড়ীতে (In the house).. 1
সাব-সেন্টারে (Sub-centre)____________________ 2
মতলব স্বাস্থ্য কেন্দ্রে (Matlab ICDDR,B)................. 3
থানা হেল্‌থ কম্‌প্লেক্স (THC).................................... 4
জেলা সদর হাসপাতাল (District Hospital)................ 5
অন্যান্য, উল্লেখ করুন (Other, specify) 6
__________________________________|___|___|
এফডবিউসি (FWC)... 7
জানিনা (Don't know)... 9

MB05. আপনার বাচ্চার জন্মের সময় কেকে ছিল যারা বাচ্চার জন্মের ব্যাপারে সাহায্য করেছে? [*প্রোব করে যারা বাচ্চার জন্মের সময়ে ছিল তাদের নাম জেনে নিন এবং নির্দিষ্ট কোড বৃত্তায়িত করুন*]

Please tell me all who were present at the time your baby was born and helped with the delivery? [*please probe to get a full list and then circle all*]

নিজে (Self).. 1
আনট্রেইন্ড আত্মীয়/প্রতিবেশী (Un-trained relative/neighbour)........ 2
আনট্রেইন্ড দাই (Untrained Dai)... 3
ট্রেইন্ড আত্মীয়/প্রতিবেশী (Trained relative/neighbour) 4
ট্রেইন্ড দাই (Trained Dai) 5
সি এইচ আর ডবিউ (CHRW).. 6
মিডওয়াইফ/নার্স (Midwife/Nurse).. 7
ডাক্তার (Doctor).. 8
অন্যান্য (Other)__________________________________...... 9

MB06. কিভাবে আপনার বাচ্চার জন্ম হয়েছে? বাচ্চার শরীরের কোন অংশ প্রথমে বের হয়েছে?

How did the baby come out and which part came out first?

সিজারিয়ান করে (Caesarian) ....1 (MB08 প্রশ্নে যান/Go to MB08)
ভ্যাজাইনাল, মাথা প্রথমে (Vaginal, head first)......................2
ভ্যাজাইনাল, কোমর প্রথমে (Vaginal, breech first)................3
ভ্যাজাইনাল, মুখ প্রথমে (Vaginal, face first) 4
ভ্যাজাইনাল, হাত/কাঁধ প্রথমে... 5
(Vaginal, hand or shoulder first)
ভ্যাজাইনাল, পা প্রথমে (Vaginal, legs first) 6
ভ্যাজাইনাল, নাড়ি প্রথমে (Vaginal, cord came out first).......7
ভ্যাজাইনাল, প্রথমে কি এসেছে জানিনা 8
(Vaginal, don't know which part came first)

MB07. ভ্যাজাইনাল হলে কি নীচের কোনটি করা হয়েছিল? (If vaginal, was)

	না **(No)**	হ্যাঁ **(Yes)**	জানিনা **(Don't know)**
এপিসিওটমী (Episiotomy done)	1	2	9
ভ্যাকিউম এক্সট্র্যাকশান (Vacuum extraction used)	1	2	9
স্যালাইনের মধ্যে ইন্জেকশন (Injection in saline infusion used)	1	2	9

MB08. আপনার বাচ্চার জন্মের সময় কি আপনার নীচের সমস্যাগুলোর কোনটা হয়েছিল?
Did any of the following problems occur during childbirth?

	না **(No)**	হ্যাঁ **(Yes)**	জানিনা **(Don't know)**
অতিরিক্ত রক্তক্ষরণ (Excessive bleeding)	1	2	9
ফুল বের না হওয়া (Retained placenta)	1	2	9
জ্বর (Fever)	1	2	9
খিচুনী (Convulsion)	1	2	9
নাড়ি বাচ্চার গলায় প্যাচানো (Cord around neck of baby)	1	2	9

MB09. **মায়ের শারীরিক পরীক্ষা** (Physical examination of mother):

Systolic BP |____|____|____| mm of Hg

Diastolic BP |____|____|____| mm of Hg

মুখের তাপমাত্রা (Oral Temperature): ...... |____|____| . |____| ºC

	**Absent**	**Present**	**Refused**
যোনী পথে রক্তক্ষরণ (Vaginal bleeding)	1	2	7
যোনী পথ ছিঁড়ে যাওয়া (Vaginal tear))	1	2	7
সেলাই ছাড়া এপিসিওটমী (Un-sutured episiotomy))	1	2	7
যোনী পথ দিয়ে দুর্গন্ধযুক্ত তরল বের হয়েছে) (Foul-smelling vaginal discharge)	1	2	7

[কোন স্বাস্থ্য সমস্যা থাকলে প্রদত্ত গাইডলাইন অনুযায়ী *মতলব হাসপাতালে পাঠান]* **[Please use attach guidelines to refer women with health problems to Matlab]**

বাচ্চার অংশ (Neonatal):

বাচ্চা যদি খুব অসুস্থ হয় তাহলে তাকে যত তাড়াতাড়ি সম্ভব রেফার করুন। সেই ক্ষেত্রে এই ফর্মটি পরে পুরণ করতে হবে। বাচ্চাকে রেফার করার জন্যে সাহায্য করুন।

[If the child is very sick - please refer immediately. The form can be completed later. Please help with transportation]

MB50. নবজাতক কি পুত্র না কন্যা? Is the baby a boy or a girl?

পুত্র (Boy) 1
কন্যা (Girl) 2

MB51. আপনার বাচ্চা কেমন আছে? How is your baby now?

সুস্থ (Healthy) .... 1 (MB53 প্রশ্নে যান/Go to MB53)
অসুস্থ (Not healthy) .... 2 (MB53 প্রশ্নে যান/Go to MB53)
বাচ্চা মারা গেছে (Died) 3

MB52. জন্মের কতক্ষন পর বাচ্চা মারা গেছে? How long after birth did the baby die?

জন্মের ....|___|___| ঘন্টার মধ্যে (within hours of birth)

MB53. জন্মের সময় আপনার বাচ্চার রং কেমন ছিল? What was the colour of the baby at birth?

স্বাভাবিক (Normal).. 1
নীল্‌চে (Blue).. 2
ফ্যাকাসে বা সাদা (Pale or white)........................... 3

MB54. জন্মের পর আপনার বাচ্চা কি নিজে থেকে কেঁদেছিল? Did the baby cry spontaneously after birth?

না (No) 1
হ্যাঁ (Yes) 2

MB55. জন্মের পর আপনার বাচ্চা কি শ্বাস-প্রশ্বাস নিয়েছিল বা নড়াচড়া করেছিল?
Did the baby spontaneously breath or show any movement after birth?

না (No).. 1
হ্যাঁ (Yes) 2

MB56. জন্মের পর আপনার বাচ্চাকে কাঁদানোর জন্যে কি কিছু করা হয়েছিল? 'না' হলে MB58 প্রশ্নে যান যদি 'হ্যাঁ' হয় তাহলে কি করা হয়েছিল? [সবগুলো উত্তর বৃত্তায়িত কর" না॥ (Was anything done to make the baby cry or move? If no, Go to ques MB58 if yes, what?) [*circle all reported]*

পিঠ চাপরানো (Slapped on back) 1
বাচ্চাকে ঝাঁকানো (Shaked the baby) 2
মুখ থেকে মুখে শ্বাস-প্রশ্বাস (Mouth to mouth respiration) 3
বাচ্চার গায়ের উপর পানি ঢালা (Poured water on the baby).............. 4
বাচ্চার মাথা নীচের দিকে ধরা Turned upside down.......................... 5
অন্যান্য (Other).. 7 |____|____|
(____)

MB57. এসব করার পর আপনার বাচ্চা কি কেঁদেছিল? Were you able to make the baby cry by doing this?

হ্যাঁ, খুব সহজেই কেঁদেছিল 1
Yes, it was very easy to make the baby cry
হ্যাঁ, চেষ্টা করার কিছু সময় পরেই কেঁদেছিল 2
Yes, after a while we managed to make the baby cry or move
না, চেষ্টা করেও বাচ্চাকে কাঁদাতে বা নড়াচড়া করাতে পারিনি বা
জীবনের কোন লক্ষন দেখা যায়নি 3
No, we did not manage to make the baby cry or make any movement or show any signs of life at all

MB58. *বাচ্চা কি এখন জীবিত আছে? - MB51প্রশ্ন মিলিয়ে দেখুন। Is the baby alive now – check MB51*

না (No) .... 1 (থামুন/STOP)
হ্যাঁ (Yes) 2

MB59. বাচ্চাকে কি এখন বুকের দুধ খাওয়ায়েছেন? [যদি না খাওয়ানো হয় তাহলে প্রোব করে জেনে নিন বাচ্চাকে বুকের দুধ দেওয়া হয়েছে কিনা] Is the baby suckling/breastfeeding? [*If not - probe if breast milk is being offered to baby]*

না (No) 1
হ্যাঁ (Yes) 2
বাচ্চাকে বুকের দুধ দেওয়া হয়েছে না 8
Breast milk not given to baby

MB60. বাচ্চাকে বুকের দুধ ছাড়া অন্যকিছু খেতে বা পান করতে দেওয়া হয় কি?
Are you giving anything else to eat or drink to your baby?

না (No)... 1 (MB63 প্রশ্নে যান/Go to MB63)
হ্যাঁ (Yes) 2

MB61. যদি 'হ্যাঁ' হয় তাহলে বাচ্চাকে বুকের দুধ ছাড়া আর কি কি দেওয়া হয়? (সবগুলো উত্তর বৃত্তায়িত করুন)
If so, what else are you giving to your baby? [*circle all reported]*

সাদা পানি (Plain water)....................................... 1
চিনি/মিশ্রির পানি Sugar water/Misri water.............. 2
মধু (Honey) 3
সরিষার তেল (Mustard oil).................................. 4
বুকের দুধ বাদে অন্য দুধ 5
(milk other than breast milk)
অন্য কোন তরল (Other liquid)________________6
নরম থকথকে খাবার (Semi-solid/gruel) 7

MB62. আপনি বাচ্চাকে এসব খাবার কিভাবে খাওয়ান? (সবগুলো উত্তর বৃত্তায়িত করুন)
How do you feed these to the baby? [*circle all reported]*

চামচ দিয়ে (Spoon).. 1
কাপ দিয়ে (Cup).. 2
বোতল ও নিপল দিয়ে (Bottle with nipple) 3
অন্যান্য (Other)___________________________7

MB63. **বাচ্চার শারীরিক পরীক্ষা** (Physical examination of the baby):

	No	Yes
a. *জিজ্ঞাসা করুন:* বাচ্চার কি খিচুনী হয়েছিল?	1.........	2

Ask: Has the infant had convulsions?

b. *জিজ্ঞাসা করুন:* বাচ্চার কি শ্বাসকষ্ট আছে? .... 1.......... 2
Ask: Does the infant have difficulty in breathing?

c. *গুনে দেখুন:* ১ মিনিটে শ্বাস-প্রশ্বাসের সংখ্যা .... ______/মিনিট
Count: the breaths in one minute
আবার গুনুন যদি >৬০/মিনিট হয় (Repeat if >60/min) .... ______/মিনিট

No **Yes**

d. *দেখুন:* বাচ্চা কি নিস্তেজ বা অজ্ঞান বা স্বাভাবিকের চেয়ে কম নরাচড়া করছে? . 1.......... 2
Look: Baby is lethargic or unconscious or has less than normal movements

e. *দেখুন এবং অনুভব করুন:* মাথার তালু ফুলেছে কিনা? .... 1.......... 2
Look and feel: Bulging fontanelle present

f. *দেখুন ও শুনুন:* ঘরঘর শব্দ আছে?.. 1.......... 2
Look and listen: Grunting present

g. *দেখুন:* বুকের খাচা মারাত্মক ভাবে বসে যায় কিনা .... 1.......... 2
Look: Severe chest indrawing present

h. *অনুভব করুন:* বাচ্চার কি জ্বর আছে বা শরীর কি অস্বাভাবিক শীতল............. 1.......... 2
Feel: Does the infant have fever or feels unusually cool

i. *দেখুন:* বাচ্চার চামড়ায় কি পুঁজ সহ দানা আছে?
Look: Are there skin pustules on the infant's skin:
না (No).. 1
হ্যাঁ, কিছু পুঁজ সহ দানা আছে (Yes, some skin pustules)................. 2
হ্যাঁ, অনেক বা মারাত্মক পুঁজ সহ দানা আছে....................................... 3
(Yes, many or severe skin pustules)

No **Yes**

j. *দেখুন:* চোখ থেকে পুঁজ বের হয়েছে কিনা? .... 1.......... 2
Look: Pus discharging from eyes

k. *দেখুন:* নাভি লালচে বা নাভি থেকে পুঁজ বের হয়েছে কিনা
Look: Umbilicus is red or draining pus:
না (No).. 1
হ্যাঁ, নাভি লালচে বা নাভি থেকে পুঁজ বের হয়েছে তবে
নাভির চারদিকের চামরা লাল নয়... 2
(Yes, is red or draining pus but redness does not extend to skin)
হ্যাঁ, নাভি লালচে বা নাভি থেকে পুঁজ বের হয়েছে এবং
নাভির চারদিকের চামরা লালচে... 3
(Yes, is red or draining pus but redness extends to skin)

l. *দেখুন:* হাত বা পায়ের পাতা কি হলদে?
Look: Palm or soles are yellow:
না (No).. 1
হ্যাঁ, অল্প হলদে ভাব (Yes, mild yellow colouration).............. 2
হ্যাঁ, মারাত্মক জন্ডিস (Yes, significant jaundice).................... 3

[উপরের যে কোন লক্ষন পাওয়া গেলে তাড়াতাড়ি মতলব হাসপাতালে পাঠান। তবে যদি শুধু i, k বা l এর কোন একটিতে "2" কোড হয় তাহলে সাব সেন্টারে পাঠান] [If any sign or symptom is positive refer immediately to Matlab; but if only "2" code found in i, k or l then refer to SC]

বগলের তাপমাত্রা (Axillary Temperature): .... |____|____| . |____| °C

ওজন (Weight):.. |____|____|____|____| g

ব্যবহৃত স্কেলের নাম লিখুন (Write the name of scale used)

দৈর্ঘ্য (Length-Left): .... |____|____| . |____| cm

(Length-right) .... |____|____| . |____| cm

মাথার পরিধি (Head circumference): .... |____|____| . |____| cm

বুকের পরিধি (Chest circumference):............. |____|____| . |____| cm

ওজন, দৈর্ঘ্য, ইত্যাদি মাপার তারিখ): .... ______/______/______
(Date of measuring weight, length, etc)

[বাচ্চার তাপমাত্রা 37.5 °C-এর বেশী বা 35.5 °C-এর কম হলে তাড়াতাড়ি মতলব হাসপাতালে পাঠান]
[If infant's temperature is more than 37.5 °C or is below 35.5 °C refer immediately to Matlab]

MB64. বাচ্চার কোন সমস্যা আছে কিনা (ক্লেফ্ট লিপ/প্যালেট, ক্লাব ফুট ইত্যাদি)
Any abnormality in the baby (cleft lip/palate, club foot, etc.), if yes, what:

না (No)... 1
হ্যাঁ (Yes)_____________________________2 |____|____|

[বাচ্চার ওজন ১,৫০০ গ্রামের কম হলে বা তার গঠনে কোন সমস্যা থাকলে তাড়াতাড়ি মতলব হাসপাতালে পাঠান]
[If infant weighs less than 1,500g or if there is any abormality refer immediately to Matlab]

MB65. *বাচ্চা কি আই সি ডি ডি আর,বি সাব-সেন্টার বা হাসপাতাল বাদে অন্য কোন হাসপাতাল/ক্লিনিকে হয়েছে?*
Was the baby born in any hospital or clinic other than ICDDR,B sub-centre/hospital?

না (No)... 1 (থামুন/STOP)
হ্যাঁ (Yes) 2

MB66. *সেই হাসপাতাল/ক্লিনিকে বাচ্চার কি ওজন নেয়া হয়েছিল? ওজন নেয়া হয়ে থাকলে কত ওজন হয়েছিল তা কি মা'র বা পরিবারের অন্য কারও কি মনে আছে?*
Was the baby weighed in that hospital or clinic? If weighed, does the mother or anyone else in the family remember the weight?

ওজন নেয়া হয়নি (Not weighed) .... 1 (থামুন/STOP)
ওজন নেয়া হয়েছিল তবে কারও মনে নেই............... 2 (থামুন/STOP)
(Weighed, but nobody remembers the weight)
ওজন নেয়া হয়েছিল এবং তা মনে আছে 3
(Weighed, and weight is known)

MB67. হাসপাতাল/ক্লিনিকে বাচ্চার ওজন কত ছিল? How much did the baby weigh in the hospital or clinic?

a1. ওজন-কিলোগ্রামে (Weight in kilograms): ....|____|.|____| kg
a2. ওজন-পাউন্ডে (Weight in pounds):.........................|____|.|____| lb

b. ওজন নেয়ার তারিখ: .... ______/______/______
(Date of measuring weight)
