# Supplementary material for: First-trimester fetal growth restriction and the occurrence of miscarriage in rural Bangladesh: A prospective cohort study
Source: PLoS One. 2017 Jul 21;12(7):e0181967. doi: 10.1371/journal.pone.0181967 (PMC5521847; doi:10.1371/journal.pone.0181967)
Supplement: S1 Table — (PDF) [file pone.0181967.s004.pdf]

S1 Table. Distribution of gestational age at CRL measurement and follow-up time of the study participants in the cohort, n=3058

	Pregnancies with miscarriage n=92 (%)	Continued Pregnancies n=2966 (%)
Gestational age at CRL measurement (wk)		
6 to 8	63 (68.5)	1437 (48.4)
9 to 11	27 (29.3)	1352 (45.6)
12 to <14	2 (2.2)	177 (6.0)
Cohort follow-up time (wk)		
0 to 5	67 (72.8)	0
6 to 10	24 (26.1)	0
11 to 15	1 (1.1)	1 (<0.0)
16 to 20	0	19 (0.6)
21 to 25	0	129 (4.3)
26 to 30	0	1657 (55.9)
31 to 35	0	1151 (38.8)
36 to 37	0	9 (0.3)

Abbreviation: GA; gestational age; wk; week.
